# Microbiology and Climate Change: a Transdisciplinary Imperative

**DOI:** 10.1128/mbio.03335-22

**Published:** 2023-02-01

**Authors:** J. T. Lennon, S. D. W. Frost, N. K. Nguyen, A. L. Peralta, A. R. Place, K. K. Treseder

**Affiliations:** a Indiana University, Bloomington, Indiana, USA; b Microsoft Health Futures, Redmond, Washington, USA; c London School of Hygiene and Tropical Medicine, London, United Kingdom; d American Society for Microbiology, Washington, DC, USA; e East Carolina University, Greenville, North Carolina, USA; f University of Maryland Center for Environmental Science, Baltimore, Maryland, USA; g University of California Irvine, California, USA; Georgia Institute of Technology

**Keywords:** biodiversity, climate change, global change, industry, infectious disease, interdisciplinary, microbial ecology, policy, transdisciplinary

## Abstract

Climate change is a complex problem involving nonlinearities and feedback that operate across scales. No single discipline or way of thinking can effectively address the climate crisis. Teams of natural scientists, social scientists, engineers, economists, and policymakers must work together to understand, predict, and mitigate the rapidly accelerating impacts of climate change. Transdisciplinary approaches are urgently needed to address the role that microorganisms play in climate change. Here, we demonstrate with case studies how diverse teams and perspectives provide climate-change insight related to the range expansion of emerging fungal pathogens, technological solutions for harmful cyanobacterial blooms, and the prediction of disease-causing microorganisms and their vector populations using massive networks of monitoring stations. To serve as valuable members of a transdisciplinary climate research team, microbiologists must reach beyond the boundaries of their immediate areas of scientific expertise and engage in efforts to build open-minded teams aimed at scalable technologies and adoptable policies.

## PERSPECTIVE

Microorganisms play a crucial role in climate change. For billions of years, they have transformed our planet, ensuring the persistence of life as we know it by regulating the composition of gases in Earth’s atmosphere. Yet microorganisms are sensitive to the effects of climate change, such as rising temperatures, increasing drought, and decreasing pH and oxygen levels in the global oceans. Some of these changes may lead to the redistribution of existing pathogens or favor the emergence of new infectious diseases that will demand attention from the public health sector, especially as human populations become more densely packed into urban ecosystems. The complexity of the microbial biosphere and its interactions with plant and animal hosts poses major challenges when it comes to predicting and mitigating the effects of climate change. However, opportunities exist to “micromanage” microbes in ways that may help to solve problems associated with climate change (1).

In the 21st century, microorganisms are being studied in previously unimaginable ways. High-throughput technologies are generating meta-genomic, -transcriptomic, and -proteomic data from natural, engineered, and host-associated ecosystems across the globe. Increasingly, we can identify the origins and distributions of traits among microorganisms, which can be used to inform us about their biogeography and functioning in the wild. Combined with advances in molecular genetics and imaging, we have a more detailed understanding of microbial physiology, metabolism, and ecology than ever before. How can we best use this information to address the climate crisis?

To make meaningful and timely progress, we contend that microbiologists must take active measures to work with people outside their traditional disciplines. In this essay, we present three case studies illustrating how a transdisciplinary approach provides critical insight for addressing the role of microorganisms in climate change. We also discuss how new perspectives on training and collaboration are needed to build diverse teams so that adoptable technology and policy can be successfully implemented. Such efforts are urgently needed to develop large-scale climate solutions.

## TRANSDISCIPLINARY APPROACHES FOR MICROBIOLOGISTS ADDRESSING CLIMATE CHANGE

### Case study 1: climate change and emerging infectious diseases.

**FIG 1:**
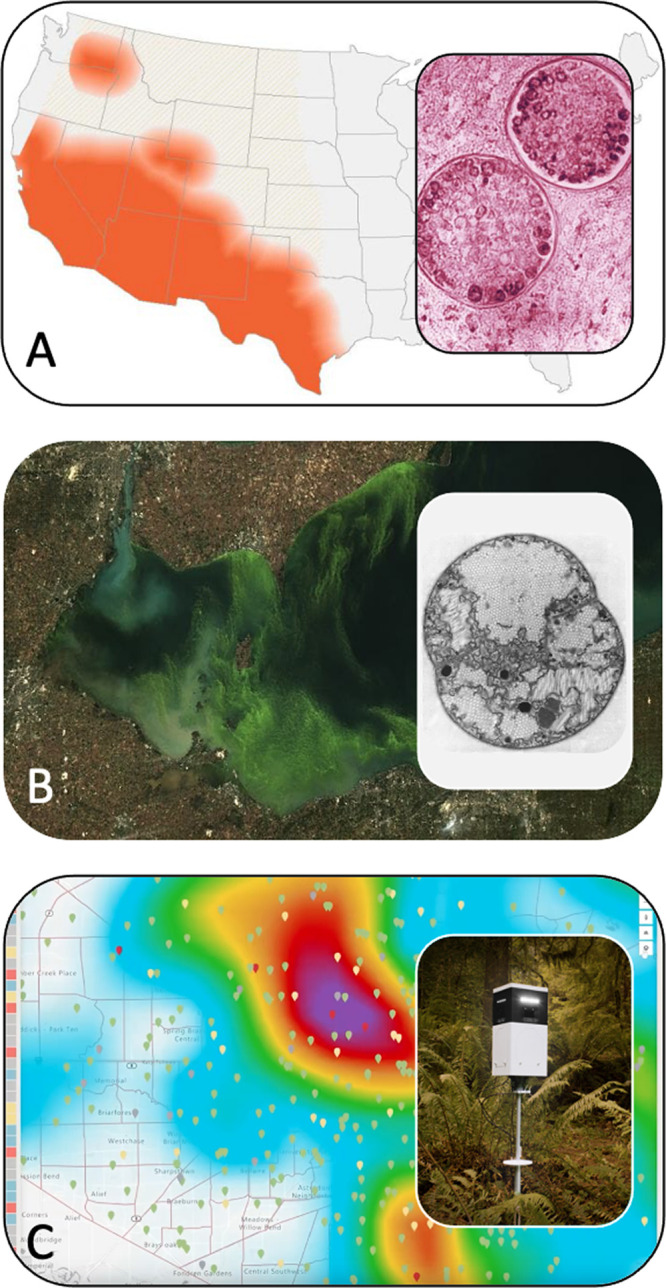
Transdisciplinary approaches to microbiology and climate change. (A) Microbial ecologists, climate modelers, and public health researchers are predicting how climate change will affect the distribution of the emerging fungal pathogen *Coccidioides*, which causes valley fever. Map showing distribution of valley fever (photo credit: CDC). Inset: spherules of *Coccidioides* containing endospores (photo credit: CDC/Lucille K. Georg). (B) Microbiologists and lake managers are taking a trait-based approach that uses ultrasonic radiation to disrupt the gas vesicles of cyanobacteria which contribute to harmful algal blooms. Satellite image of bloom (photo credit: Richard Stumpf, NOAA, derived from NASA MODIS data). Inset: cross-section of bloom-forming cyanobacterium, *Microcystis*, with light-colored, hexagonal vesicles. (Micrograph by H. S. Pankratz, republished from Walsby [7] with permission.) (C) Researchers are massively deploying sensors to monitor and integrate climatic and biological data to predict changes in the abundance and distribution of disease-causing microorganisms and their insect vectors. Map of Harris County, TX, which includes the metropolitan Houston area. Warmer colors depict areas with elevated precipitation, which can increase the risk of West Nile virus; symbols represent sensor locations (photo credit: Microsoft, Inc.). Inset: sensor used as part of “Computing the Biome” project (photo credit: Microsoft, Inc.).

The US CDC has described coccidioidomycosis (valley fever), a pneumonia-type disease caused by fungi in the genus *Coccidioides*, as a “silent epidemic” in the southwestern United States (Fig. 1A). Patients contract the disease by inhaling spores of these fungi from the environment; it is seldom passed person-to-person. The fungus seems to prefer warmer temperatures, grows when water is available, and forms spores as soils dry. These spores then become airborne as winds scour the soil surface. Cases of valley fever have been rising over the past 20 years. In California, valley fever increased by >200% between 2014 and 2018 (2). Climate change is likely contributing to this upward trend (3).

A transdisciplinary research team recently addressed the question of whether climate change influences the spread of valley fever. The team required expertise in microbial ecology, epidemiology, and earth system modeling. Earth system models were a critical component because they simulate large-scale climate dynamics and can map the rate and magnitude of climate change in future centuries. It is still rare for microbiologists to collaborate with earth system modelers, yet this approach was required to predict the future spread of valley fever under climate change. Most earth system models do not explicitly represent microorganisms in their simulations, in part to a dearth of data regarding microbial species’ geographical distribution and ecological niches (4). The challenge was linking the temporal and geographical distribution of *Coccidioides* to environmental factors and then using the earth system model to identify where and when *Coccidioides*’ preferred environments will occur as climate change proceeds.

Because it is difficult to detect *Coccidioides* in the environment due to their low incidence and our lack of understanding of where they reside within the soil, the research team focused on the epidemiology of valley fever (5). Together, they found that outbreaks occur primarily in areas where the mean annual temperature exceeds 16°C and mean annual precipitation is less than 600 mm (5). In this way, the research team was able to define the climate niche of *Coccidioides*. Next, they parameterized an earth system model with the climate niche (3). This model predicted that climate change within this century will expand the geographic range that falls within the climate niche of *Coccidioides* (3).

Under current trajectories of climate change, valley fever is predicted to expand northward and become endemic in every western US state by the end of the century (3). These predictions allow public health agencies to prepare for outbreaks in new populations by informing doctors to watch for valley fever symptoms, communicating health alerts to outdoor workers, and requiring mandatory reporting in the new areas of endemicity. This societal adaptation to climate change-induced disease spread would not have been possible without the expertise of microbial ecologists, climate modelers, and public health researchers.

### Case study 2: multi-stakeholder approach to water quality and climate change.

Climate change is altering microbial communities in ways that threaten sustainable water supplies throughout the world. There are very few freshwater ecosystems, including the Great Lakes, that are not affected by cyanobacterial blooms (Fig. 1B). More than a decade ago, it was predicted that climate change would lead to the global expansion of harmful cyanobacterial blooms (6). Notably, the spread of bloom-causing species poses serious health concerns while threatening ecosystem services, including fishing, irrigation, and drinking water supplies (6).

To mitigate harmful blooms, transdisciplinary teams were established to target unique traits of cyanobacteria. Understanding the biological regulation of cyanobacteria is the critical first step. It is well known that many species of cyanobacteria regulate their buoyancy in the water column using internal structures known as gas vesicles (Fig. 1B) (7). These cyanobacteria gather at the surface during the day to harvest light for photosynthesis, shading out other phytoplankton lower in the water column. Later in the day, accumulated carbohydrates from daytime photosynthesis overcome the buoyancy provided by the gas vesicles, and the cells sink to cooler, nutrient-rich water. The ability to regulate their vertical position in the water column allows gas vesicle-containing cyanobacteria to maintain dominance over other non-blooming phytoplankton (7). The continued rise in nutrients, temperature, and CO_2_ may exacerbate the spread and persistence of harmful cyanobacteria blooms.

Transdisciplinary microbiology has the potential to manage cyanobacterial blooms and ensure healthy water supplies. One promising solution is using ultrasound radiation to reduce cyanobacterial growth by causing the collapse of the gas vesicles, resulting in a loss of buoyancy and cell sedimentation (8). In the laboratory, effective removal of most harmful cyanobacterial bloom-forming species may occur in as little as 10 min of exposure. Based on such results, several commercial instruments have been developed to deploy ultrasound technology in natural settings. Initial assessments suggest that economic benefits to local communities can be observed in less than 2 years after applying this ultrasound technology (9). Additional studies and optimization are needed to support the scaling and application of this approach and to evaluate its potential non-target effects on other aquatic organisms. This case study demonstrates how transdisciplinary initiatives involving trait-based microbiology, engineering, and aquatic sciences have the potential to restore ecosystems and the services that they provide to local communities (10).

### Case study 3: microbe-driven solutions on a global scale.

Natural and managed ecosystems are complex, characterized by high levels of uncertainty, especially when forced by environmental drivers associated with climate change. To better capture this variability, researchers rely on new technologies that can be used to understand the connection between climate change and microbial processes. One such approach involves the deployment of sensors to generate spatial and temporal data that will aid in modeling and decision making. For example, “Computing the Biome” is a program that uses sensor-collected data along with an artificial intelligence platform to predict biothreats (11). This framework interconnects novel data streams ranging from kilometer-scale hyper-local weather, to autonomously identified disease-transmitting insects that may only be millimeters in size, to both known and novel viruses that are only nanometers in size.

To achieve these aims, a transdisciplinary and multisectoral team was assembled with combined expertise in ecology, epidemiology, and virology from academic institutions, public health agencies, and industry. Their initial goal was to predict mosquito-borne disease in Harris County, TX, with the long-term vision of establishing a global network of “biological weather stations” that provides continuous, high-resolution data about viruses, microbes, and arthropods, which can inform decision-making and policy (Fig. 1C).

The combination of multiple data streams and expertise in a network can help prioritize when and where to sample. By directly addressing a need (an early warning system for mosquito-borne disease) and making it cost-effective (inexpensive digital sensing guiding biological sampling), this infrastructure can be more economically sustainable and be used for other purposes. Personnel employed to visit field sites and maintain devices can collect data relevant to multiple disciplines. Once established, this infrastructure can be used to accommodate other data sets, including spatial information and sequence data along with the associated bioinformatic pipelines.

## OPPORTUNITIES AND CHALLENGES FOR TRANSDISCIPLINARY MICROBIAL CLIMATE-CHANGE RESEARCH

To serve as valuable members of a transdisciplinary climate research team, microbiologists must reach beyond the boundaries of their immediate areas of scientific expertise. In this section, we highlight challenges, opportunities, and skillsets that will advance the integration of microbiology into climate change research and policy.

### Scaling microbial processes to climate solutions.

Microorganisms can be leveraged to enhance food security, water supplies, and air quality in a changing world. However, insights gained across scales—spanning single cells to whole ecosystems—requires thoughtful design and intentional management. Microbiologists must wrestle with how variation in the ecological and evolutionary processes associated with diverse microbial life forms, such as horizontal gene transfer, dispersal, and functional redundancy, affect decisions and policy of practical importance. Ultimately, microbiologists must strive to make connections so that their discoveries can be used to inform allied disciplines and contribute to larger scale climate-change solutions.

### Building, cultivating, and sustaining creative and diverse teams.

Building effective teams takes effort and patience. It is even more difficult when team members are from different disciplines or sectors, or have limited scientific background. The importance of integrating diverse perspectives is an important skillset that requires attention and reinforcement in traditional STEM training programs. Increased funding for developing and implementing training programs will build capacity for transdisciplinary training. Forums are needed to break down barriers associated with discipline-specific jargon. Moreover, diverse teams require time to build trust before members are ready to co-produce adoptable climate solutions (12). Transdisciplinary collaborations would benefit from long-term agency support with different expectations of what ‘products’ may look like. Even after developing and testing microbially focused climate solutions, scaling them within established fields requires a different set of knowledge and relationships, not to mention time.

### Microbiology and climate change policy.

Ultimately, microbiologists are needed to inform climate change research policies to address environmental justice and achieve health equity (13). Working in the policy space presents a unique set of challenges, especially for scientists. It requires sufficient knowledge of the political system, including opportunities for complementary goals and competing interests for implementation. It also requires a strong partnership with experts to navigate through the policy landscape in order to integrate research findings into practical policy recommendations. Thus, microbiologists need to participate in policy conversations, engage with policymakers at the local and federal levels, and consider the implications of their research to advocate for evidence-based policies to address climate change effectively.

## CONCLUSIONS

In microbiology and other fields, conventional scientific training tends to emphasize in-depth expertise in a single discipline. With the increasing complexity and urgency of societal problems such as climate change, new approaches and mindsets are needed to create novel solutions. This requires the scientific community to go beyond any one discipline or sector, unifying intellectual frameworks from different fields and generating new perspectives and knowledge. We contend that a transdisciplinary approach is no longer optional but a must-do approach to address the complex issues of climate change.

More than ever, microbiologists are in a position to lead or take active roles in transdisciplinary climate research teams. To be effective in this capacity, microbiologists need to adopt an open mindset, acquire additional skills, and collaborate with new partners, including social scientists, engineers, and policy makers. Fortunately, advancements in education, communication, and management will support the implementation of a transdisciplinary approach. The field of microbiology is still observing the dawn of this transformation. Initial evidence is promising, and the potential outcomes are rewarding.
